# Fhit expression in human gastric adenomas and intramucosal carcinomas: correlation with Mlh1 expression and gastric phenotype

**DOI:** 10.1038/sj.bjc.6601601

**Published:** 2004-02-03

**Authors:** K Kawaguchi, K Yashima, M Koda, A Tsutsumi, S Kitaoka, H Andachi, A Hosoda, Y Kishimoto, G Shiota, H Ito, Y Murawaki

**Affiliations:** 1 Division of Medicine and Clinical Science, Faculty of Medicine, Tottori University, Yonago 683-8504, Japan; 2 Division of Organ Pathology, Faculty of Medicine, Tottori University, Yonago 683-8504, Japan; 3 Division of Pharmacotherapeutics, Faculty of Medicine, Tottori University, Yonago 683-8504, Japan

**Keywords:** gastric cancer, Fhit, Mlh1, mucin phenotype, immunohistochemistry, endoscopic mucosal resection

## Abstract

The *fragile histidine triad* (*FHIT*) gene, encompassing the FRA3B fragile site at chromosome 3p14.2, is a candidate tumour suppressor gene involved in a variety of tumours, including gastric carcinomas. Recently, it has been reported that the *FHIT* gene may be a target of damage in some of mismatch-deficient tumours. To clarify further the role of the Fhit protein in gastric carcinogenesis, we investigated whether Fhit expression in early gastric neoplasia is associated with mismatch repair protein expression and cellular phenotype. Fhit, Mlh1 and phenotypic expression were evaluated immunohistochemically in 87 early gastric neoplasias, comprising 32 adenomas and 55 intramucosal carcinomas, resected by endoscopic mucosal resection therapy. Significant loss or reduction of Fhit expression was noted in four (12.5%) of the 32 adenomas and 21 (38.2%) of the 55 intramucosal carcinomas. The rate of abnormal Fhit expression was significantly higher in intramucosal carcinomas than in adenomas (*P*=0.021). Moreover, reduced Fhit expression was found to be significantly associated with loss of Mlh1 expression in early gastric neoplasia (*P*=0.0011). Furthermore, we also detected a significant association between reduced Fhit expression and gastric phenotype (*P*=0.0018). These results suggested that reduced Fhit expression occurs in the early stage of gastric carcinogenesis and could be correlated with a lack of Mlh1 expression and gastric phenotype.

A candidate tumour suppressor gene, *fragile histidine triad* (*FHIT*), was identified at chromosome 3p14.2 spanning the FRA3B common fragile site (Ohta *et al*, 1996). Abnormal transcripts were frequently observed in a variety of human cancers including those of the digestive tract, lung, breast and head and neck (Ohta *et al*, 1996; Croce *et al*, 1999). The majority of these abnormalities include aberrant mRNA transcripts, with the absence of one or more exons within the mRNA. Genomic analysis demonstrated frequent allelic loss and homozygous deletions (Ohta *et al*, 1996; Croce *et al*, 1999).

In gastric carcinomas, alterations at the *FHIT* locus or reduced expressions of Fhit have been associated with gastric tumour progression and poor survival of cancer patients (Baffa *et al*, 1998; Capuzzi *et al*, 2000). However, conflicting results from reverse transcription (RT)–PCR analysis and genomic analysis have been reported (Ohta *et al*, 1996; Gemma *et al*, 1997; Tamura *et al*, 1997). In general, in tumours (lung and oesophagus) associated with environmental carcinogens, alterations in the *FHIT* gene occur early in cancer development, but are thought to be a late event in other cancers and possibly associated with cancer progression to more aggressive neoplasia (Croce *et al*, 1999; Kitamura *et al*, 2001). Moreover, little is known about Fhit expression in early gastric neoplasia.

Microsatellite instability (MSI) due to defects in mismatch repair (MMR) genes such as *MLH1* and *MSH2* are involved in the carcinogenesis and tumour progression of sporadic and inherited human cancers (Eshleman and Makowitz, 1996; Kinzler and Vogelstein, 1996). Microsatellite instability is reportedly present in 15–33% of solitary gastric cancers, although mutations of the *MLH1* or *MSH2* genes are rare in sporadic gastric cancers (Chong *et al*, 1994; Mironov *et al*, 1994; Strickler *et al*, 1994; Tamura *et al*, 1996). Mismatch repair deficiency leads to the accumulation of base-base mismatches and short insertion/deletion mispairs, generated as a consequence of DNA replication errors and homologous recombinations (Eshleman and Makowitz, 1996; Kinzler and Vogelstein, 1996). Most sporadic gastric cancers with MSI have been demonstrated to be caused by somatic hypermethylation of the *MLH1* promoter region, resulting in the downregulation of *MLH1* gene expression (Fleisher *et al*, 1999,^2001^; Kang *et al*, 1999; Leung *et al*, 1999; Suzuki *et al*, 1999). Recent studies have revealed that immunohistochemistry is a reliable screening technique for identifying MMR-deficient tumours (Thibodeau *et al*, 1996; Marcus *et al*, 1999).

Recently, Fong *et al* (2000) demonstrated that *N*-nitrosomethylbenzylamine exposure caused a spectrum of visceral and skin tumours similar to Muir–Torre syndrome, caused by deficiency in an MMR gene, in Fhit-deficient mice, and suggested that the *FHIT* gene may be a target of damage in some of MMR-deficient tumours. Moreover, Mori *et al* (2001) and Andachi *et al* (2002) reported that loss of the MMR protein is significantly correlated with the loss of Fhit expression in human colorectal carcinomas. While an association between MSI and *FHIT* alterations has been reported by Huiping *et al* (2002), the relationship between Fhit and Mlh1 expression has not been previously studied in gastric carcinomas.

Gastric carcinomas have been divided into two histological types: intestinal and diffuse types, according to Lauren (1965), or differentiated and undifferentiated types, according to Nakamura *et al* (1968). However, mucin histochemical and immunohistochemical examinations have recently demonstrated that gastric and intestinal phenotypic cell markers are widely expressed in gastric carcinomas, irrespective of their histological type (Egashira *et al*, 1999; Yoshino *et al*, 1999; Koseki *et al*, 2000). Moreover, it has generally been reported that gastric carcinomas with a predominantly gastric phenotype have a pronounced tendency toward invasion, metastasis and poor prognosis compared with gastric carcinomas that have intestinal phenotypic expression (Endo *et al*, 1999; Koseki *et al*, 1999; Yoshino *et al*, 1999; Kabashima *et al*, 2002; Tajima *et al*, 2002). Therefore, phenotypic subclassification should be useful in understanding the biologic behaviour of carcinomas and selecting a suitable therapeutic method.

To clarify the role of the *FHIT* gene in the development of gastric carcinomas, we compared Fhit expression with Mlh1 and phenotypic expression in gastric adenomas and intramucosal carcinomas.

## MATERIALS AND METHODS

### Patient samples

Tumour specimens were obtained from 87 patients (58 male and 29 female subjects), who had undergone endoscopic mucosal resection at Tottori University Hospital between 1994 and 2000. Pathologically, 32 lesions were diagnosed as adenomas and 55 lesions as intramucosal carcinomas (Table 1

**Table 1:** Clinicopathological features in early gastric neoplasia

	**Adenoma (*n*=32)**	**Intramucosal carcinoma (*n*=55)**
Gender (M : F)	18 : 14	40 : 15
Age (mean±s.d; years)	71.3±6.7	70.0±8.5
Histologic type	Mild 2	Tub1 41
(grade)	Moderate 20	Tub2 12
	Severe 10	Pap 2
*Location*		
Upper	2	4
Middle	15	20
Lower	15	31
*Gross classification*		
Elevated	22	35
Flat or depressed	10	20

Tub1=well-differentiated tubular adenocarcinoma; Tub2=moderately differentiated tubular adenocarcinoma; Pap=papillary adenocarcinoma.

). Macroscopic and histological evaluations were made according to the classification established by the Japanese Research Society for Gastric Cancer (1993). The macroscopic features were divided into two major types: elevated, and flat or depressed. The depth of invasion and histological grade were classified according to the predominant features. In this study, adenoma and intramucosal carcinoma correspond to low- or high-grade adenoma/dysplasia and noninvasive carcinoma or intramucosal carcinoma in the Vienna classification system, respectively (Schlemper *et al*, 2000). *Helicobacter pylori* infection was evaluated in 20 adenomas and 23 intramucosal carcinomas by histology, rapid urease test, bacterial culture test and serological test, and was defined by a positive result in any of these four tests. All these cases were positive. Pathological diagnoses were verified by two experienced pathologists (HA and HI). All the cases were analysed anonymously, that is, all the specimens were assigned a new number without any personal information. Institutional Review Board approval was obtained.

### Immunohistochemical staining

Paraffin-embedded, 4 *μ*m-thick sections were immunohistochemically stained with an anti-Fhit rabbit polyclonal antibody (IBL, Gunma, Japan; dilution 1 : 100), an anti-Mlh1 mouse monoclonal antibody (G168-15, PharMingen, San Diego, CA, USA; dilution 1 : 50), an anti-human gastric mucin (HGM) mouse monoclonal antibody (45M1, Novocastra Laboratories, Ltd., Newcastle, UK; dilution 1 : 50), an anti-MUC2 mouse monoclonal antibody (Ccp58, Novocastra, Newcastle, UK; dilution 1 : 100) and an anti-CD10 mouse monoclonal antibody (56C6, Novocastra, Newcastle, UK; dilution 1 : 50) using the avidin–biotin–peroxidase complex technique.

Immunohistochemical staining was performed as described below. In brief, after being deparaffinised in xylene and rehydrated in ethanol, the sections were immersed in a citrate buffer (0.01 M, pH 6.0) and heated in a microwave oven for 20–30 min to retrieve antigens, then incubated with the primary antibody overnight at 4°C. As a negative control, the primary antibody was replaced with normal serum IgG at a similar dilution. The detection reaction followed the Vectastain Elite ABC kit protocol (Vector Laboratories, Burlingame, CA, USA). Diaminobenzidine was used as a chromogen, and methylgreen or haematoxylin as a counterstain. The sections were incubated with biotinylated anti-rabbit or mouse IgG and avidin–biotin–peroxidase and visualised using diaminobenzidine tetrahydrochloride.

Then, paradoxical concanavalin A (ConA, Vector Laboratories, Burlingame, CA, USA) staining was carried out according to the method of Kabashima *et al* (2002).

The protein expression was evaluated by two independent observers (HA and KY). Immunohistochemical analysis was performed in a blinded manner with respect to the clinical information.

### Assessment of Fhit immunostaining

The Fhit expression was graded for both the extent and intensity of immunopositivity, as described previously (Hao *et al*, 2000). The extent of positivity was scored as follows: 0,<5%; 1, 5–25%; 2, 25–50%; 3, 50–75% and 4, >75% of the gastric epithelial cells in the respective lesions. The intensity was scored as follows: 0, negative; 1+, weak; 2+, moderate and 3+, as strong as normal mucosa. The final score was obtained by multiplying the positivity and intensity scores, producing a range from 0 to 12. Scores 9–12 were defined as a preserved or strong staining pattern, scores 5–8 were defined as an intermediate staining pattern and scores 0–4 were defined as markedly reduced or lost expression.

### Assessment of Mlh1 immunostaining

Normal tissue adjacent to the tumour was used as an internal positive control. The normal staining pattern for Mlh1 was nuclear. Tumour cells that exhibited an absence of nuclear staining in the presence of non-neoplastic cells with nuclear staining were considered to have an abnormal pattern. Cases with definite nuclear staining in more than 30% of the tumour cells were categorised as positive, cases with definite nuclear staining in less than 30% of the tumour cells were categorised as negative (Beak *et al*, 2001).

### Assessment of HGM, ConA, MUC2 and CD10 immunostaining and classification of the phenotypes

Human gastric mucin staining was seen in the cytoplasm of the gastric foveolar epithelium and mucous neck cells, while ConA staining was seen in the cytoplasm of the pyloric glands. MUC2 staining was seen in the cytoplasm around the nuclei of goblet cells. CD10 staining was seen along the brush border of the luminal surface of the epithelium. Although CD10 can also be expressed in the apical portion of the cytoplasm of normal gastric mucosa, only the expression of CD10 on the brush border was studied. The results of staining were categorised into two groups: positive expression and negative expression. Staining of >10% of the adenoma and carcinoma cells was classified as positive expression and <10% was classified as negative expression. The phenotypes were classified into four categories according to the combination of the expression of CD10, MUC2, ConA and HGM (Yoshino *et al*, 1999). The intestinal phenotype (I-type) demonstrated positive expression for MUC2 and/or CD10, but negative expression for both HGM and ConA. The gastric and intestinal mixed phenotype (GI-type) demonstrated positive expression for MUC2 and/or CD10, and positive expression for HGM and/or ConA. The gastric phenotype (G-type) demonstrated positive expression for HGM and/or ConA, but negative expression for both MUC2 and CD10. The unclassified phenotype (UC-type) demonstrated negative expression for MUC2, CD10, ConA and HGM.

### Statistical analysis

Statistical analysis was performed by the χ^2^ test or Fischer's exact test (two-sided). *P*<0.05 was considered significant.

## RESULTS

### Fhit expression in the normal epithelium and neoplasia of the stomach

**Figure 1:**
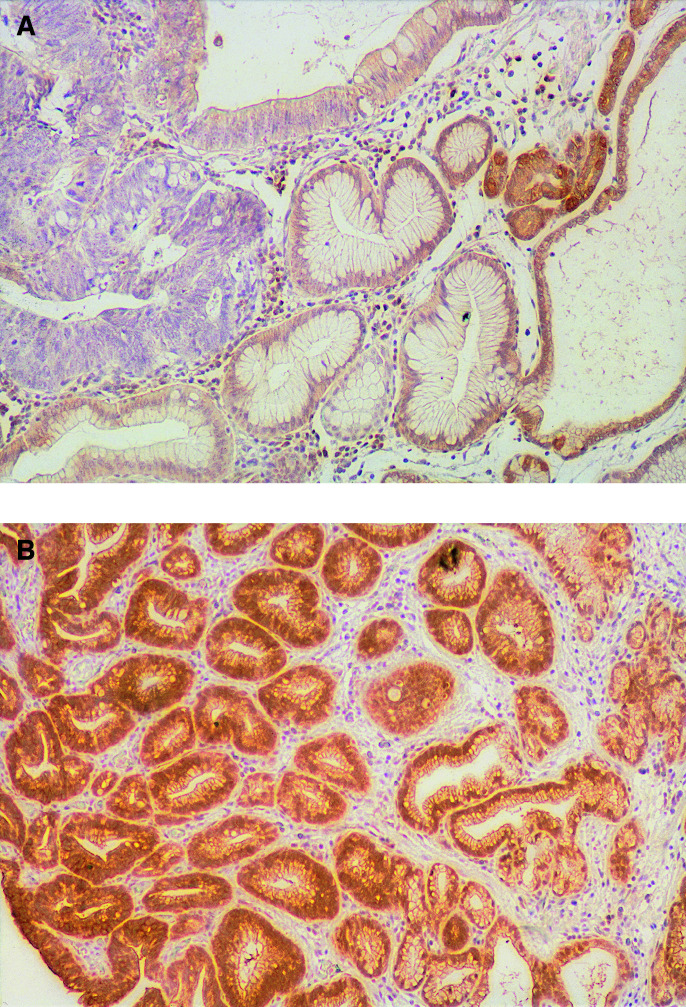
Fhit immunostaining in human gastric non-neoplastic and neoplastic tissues. (**A**) Negative immunostaining of an intramucosal carcinoma and positive immunostaining of a non-neoplastic epithelium. (**B**) Positive immunostaining of an adenomatous and non-neoplastic epithelium.

Normal gastric epithelia adjacent to the tumour cells showed moderate to strong cytoplasmic expression of the Fhit protein from the basal portion to the luminal differentiated cells; these findings served as internal positive controls. Smooth muscle cells and inflammatory mononuclear cells were positive at various intensities and degrees. Reduced or absent staining for Fhit was recognised in four (12.5%) of the 32 adenomas and in 21 (38.2%) of the 55 intramucosal carcinomas (Figure 1A and B

, Table 2

**Table 2:** Immunohistological findings in early gastric neoplasia

	**Adenoma (*n*=32)**	**Intramucosal carcinoma (*n*=55)**
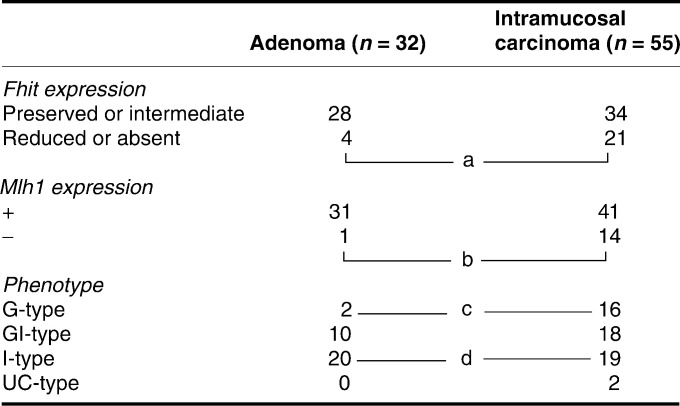		

^a^*P*=0.021, rate of abnormal Fhit expression in adenoma is lower than that in carcinoma.^b^ *P*=0.0076, rate of abnormal Mlh1 expression in adenoma is lower than that in carcinoma.^c^ *P*=0.013, rate of G-type in adenoma is lower than that in carcinoma.^d^ *P*=0.011, rate of I-type in adenoma is higher than that in carcinoma.

). The incidence of reduced Fhit expression was significantly higher in the intramucosal carcinomas than in the adenomas (*P*=0.021). However, no significant associations were found among Fhit expression and other clinicopathological parameters (data not shown).

### Mlh1 expression in the normal epithelium and neoplasia of the stomach

**Figure 2:**
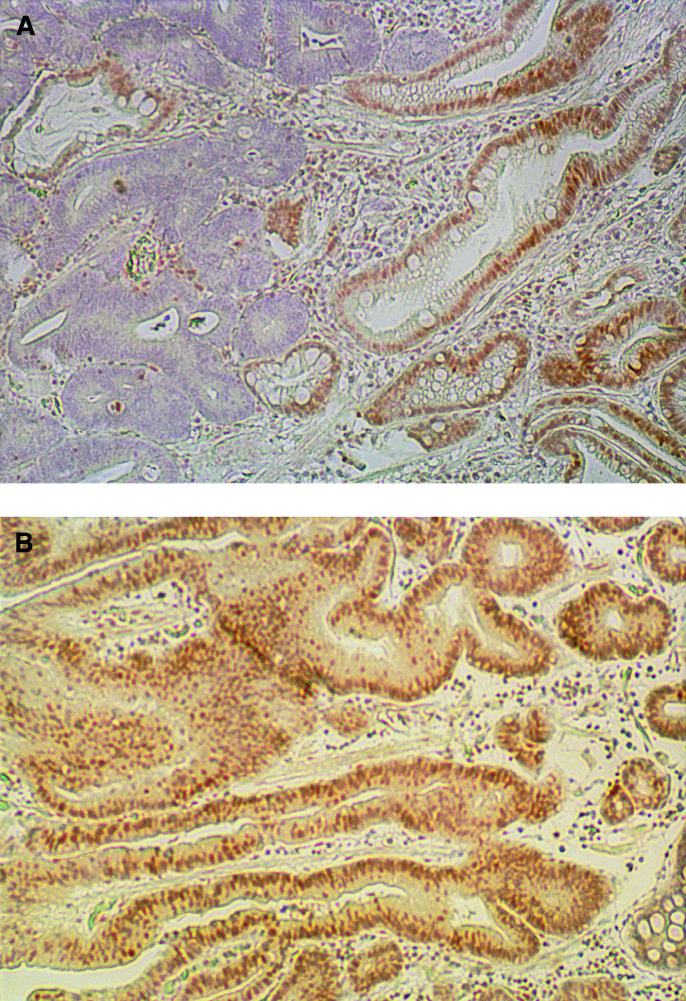
Mlh1 immunostaining in human gastric non-neoplastic and neoplastic tissues. (**A**) Negative immunostaining of an intramucosal carcinoma and positive immunostaining of a non-neoplastic epithelium. (**B**) Positive immunostaining of an intramucosal carcinoma and non-neoplastic epithelium.

The expression of Mlh1 protein was observed exclusively in the nucleus. In normal tissue adjacent to the tumour cells, Mlh1 expression was detected predominantly in the proliferative zone, such as the germinal centres of lymphoid follicles and normal glands. Normal stromal cells such as fibroblasts and endothelial cells also showed nuclear positivity for this protein. Loss of Mlh1 expression was detected in one (3.1%) of the 32 adenomas and 14 (25.5%) of the 55 intramucosal carcinomas (Figure 2A and B

, Table 2). The incidence of Mlh1 expression loss was significantly higher in the intramucosal carcinomas than in the adenomas (*P*=0.0076). However, there was no significant difference in other clinicopathological parameters (data not shown).

### Distribution of the phenotype in early gastric neoplasia

The distributions of phenotype in the adenomas and intramucosal carcinomas were two (6.3%) of 32 and 16 (29.1%) of 55 for the G-type, 10 (31.3%) of 32 and 18 (32.7%) of 55 for the GI-type, and 20 (62.5%) of 32 and 20 (36.4%) of 55 for the I-type, respectively. The frequency of the G-type in carcinomas (*P*=0.013) and the I-type in adenomas (*P*=0.011) was significantly higher than in the others (Table 2).

### Correlation of Fhit expression with Mlh1 expression in early gastric neoplasia

Among the early neoplasias with reduced or absent Fhit expression, 10 (40.0%) of 25 showed a loss of nuclear Mlh1 expression, in contrast to five (8.1%) of 62 neoplasias with preserved or intermediate Fhit expression (*P*=0.0011) (Table 3

**Table 3:** Relationship between Fhit and Mlh1 expression in early gastric neoplasia

		**Fhit expression**	
**Mlh1expression**	**No.**	**Preserved or intermediate**	**Reduced or absent**
+	72	57	15
−	15	5	10

*P*=0.0011, reduced or absent expression of Fhit is associated with loss of Mlh1.

). Regarding intramucosal carcinomas, 42.9% (nine of 21) of the reduced or absent Fhit expression carcinomas were negative for Mlh1 expression, whereas 14.7% (five of 34) of the preserved or intermediate Fhit expression carcinomas were Mlh1 negative (*P*=0.044). In the adenomas, the one case with Mlh1-negative expression was one of the four-Fhit-reduced samples.

### Relationship between phenotype and expression of Fhit and Mlh1 protein in early gastric neoplasia

Decreased or nonstaining for Fhit was detected in 11 (61.1%) of 18 G-type, eight (28.6%) of 28 GI-type and five (12.8%) of 39 I-type early gastric neoplasia (Table 4

**Table 4:** Relationship between phenotype and Fhit and Mlh1 expression in early gastric neoplasia

		**Fhit**		**Mlh1**	
**Phenotype**	**No.**	**Preserved or intermediate**	**Reduced or absent**	**+**	**−**
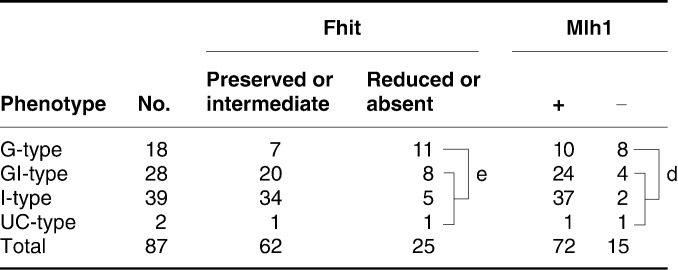					

^e^*P*=0.0018, rate of abnormal Fhit expression in the G-type is higher than that in the others.^d^*P*=0.002, rate of abnormal Mlh1 expression in the G-type is higher than that in the others.

). The incidence of reduced Fhit expression in early gastric neoplasia was significantly more frequent in the G-type compared with the others (*P*=0.0018). Regarding intramucosal carcinomas, 52.4% (11 of 21) of the reduced or absent Fhit expression carcinomas were G-type, whereas 14.7 % (five of 34) of the preserved or intermediate Fhit expression carcinomas were G-type (*P*=0.0073). Loss of Mlh1 expression was detected in eight (44.4%) of 18 G-type, four (14.3%) of 28 GI-type and two (5.1%) of 39 I-type (Table 4). The incidence of Mlh1 expression loss in early gastric neoplasia was significantly higher in the G-type than in the others (*P*=0.0021).

## DISCUSSION

Alterations and abnormal transcripts of the *FHIT* gene have been reported in a number of primary human tumours, including gastric carcinoma (Ohta *et al*, 1996; Croce *et al*, 1999). However, some of the data regarding gastric carcinoma are conflicting (Ohta *et al*, 1996; Gemma *et al*, 1997; Tamura *et al*, 1997). It was reported that alterations in the *FHIT* locus detected by DNA and/or RT–PCR analysis closely correlated with a loss of Fhit protein expression in lung and gastric carcinomas (Baffa *et al*, 1998; Sozzi *et al*, 1998). These results indicated that *FHIT* gene alteration could be detected simply by immunohistochemical analysis of tumour specimens. In this study, we observed abnormal Fhit protein expression in intramucosal carcinomas, with 38.2% of specimens demonstrating a decrease in, or absence of, Fhit protein staining. This frequency of abnormal Fhit expression was obviously low compared with that observed in advanced gastric carcinomas in previous reports (Baffa *et al*, 1998; Capuzzi *et al*, 2000). Therefore, abnormal Fhit expression may be associated with tumour progression in gastric carcinomas. However, we found no correlation between Fhit expression and any of the clincopathological parameters.

Alterations in the *FHIT* gene and its expression have been reported even in premalignant lesions of the lung, colon and oesophagus (Sozzi *et al*, 1998; Croce *et al*, 1999; Kitamura *et al*, 2001). We found a significantly reduced Fhit expression in 12.5% of gastric adenomas. However, there was no correlation between Fhit expression and the severity of histopathological changes. Caselli *et al* (2001) reported that reduced Fhit expression was not detected in precancerous lesions of the stomach. Another recent study on early gastric neoplasia revealed a higher frequency of abnormal Fhit expression comparable to studies on advanced gastric cancer (Skopelitou *et al*, 2003). These discrepancies may be caused by differences in immunohistochemical techniques, the anti-FHIT antibody used, interpretation of immunoreactivity, sample number, sample type or different distributions of histological grade. Moreover, we found that the rate of reduced Fhit expression was significantly lower in gastric adenomas than that in intramucosal carcinomas. These results suggest that Fhit protein may have a functional role in the early stage of gastric tumorigenesis.

Inactivation of MMR genes has been described as an alternative pathway in cancer development and progression (Eshleman and Makowitz, 1996; Kinzler and Vogelstein, 1996). Microsatellite instability is found in 15–33% of sporadic gastric cancers, a higher incidence than that seen in other types of sporadic human cancers (Chong *et al*, 1994; Mironov *et al*, 1994; Strickler *et al*, 1994; Tamura *et al*, 1996). Recent studies have suggested that silencing of the *MLH1* gene by promoter hypermethylation is a major causative event in the development of human gastric cancers with MSI (Fleisher *et al*, 1999,^2001^; Kang *et al*, 1999; Leung *et al*, 1999; Suzuki *et al*, 1999). The majority of these tumours also exhibited loss of Mlh1 protein expression. Thus, our finding of loss of Mlh1 expression in 14 (25.5%) of the 55 intramucosal carcinomas coincides with published frequencies. However, only one (3.1%) of the 32 adenomas showed Mlh1 expression loss. This abnormal Mlh1 protein expression was less frequent than that observed in gastric adenomas reported by other investigators (Beak *et al*, 2001). This might be partly due to different diagnostic criteria for gastric adenoma, different criteria for the loss of Mlh1 expression, varying degrees of dysplasia and the limited number of tested cases. Moreover, we indicated that the rate of reduced Mlh1 expression in gastric adenomas was significantly lower than that in intramucosal carcinomas, which is consistent with a previous report (Beak *et al*, 2001).

Recently, it has been reported that following *N*-nitrosomethylbenzylamine exposure, Fhit-deficient mice developed a spectrum of visceral and skin tumours similar to Muir–Torre syndrome, caused by deficiency in an MMR gene (Fong *et al*, 2000). A large subgroup of Muir–Torre syndrome cases exhibits MSI and germline mutations in the *MLH1* or *MSH2* gene (Kruse *et al*, 1998). In addition, it was previously observed that frequent human pancreatic cancers and cell lines with high MSI had homozygous deletions within *FHIT* (Hilgers and Kern, 1999; Hilgers *et al*, 2000). Therefore, these reports suggest that the *FHIT* gene might be a target of damage in some MMR-deficient tumours. Our data show that MMR deficiency based on the status of Mlh1 protein expression is significantly associated with reduced Fhit expression in early gastric neoplasias, supporting this hypothesis. Mori *et al* (2001) and Andachi *et al* (2002) reported an association between MMR deficiency and *FHIT* alterations in colorectal carcinomas. Moreover, as a mechanism for this, they proposed that repetitive elements, such as (CA)*n* and (A)*n* repeats, in introns 4 and 5 of the *FHIT* gene could be a target of damage in MMR-deficient tumours.

It has been reported that there are obvious differences in the biological behaviour of gastric phenotype carcinomas and intestinal phenotype carcinomas (Egashira *et al*, 1999; Yoshino *et al*, 1999; Koseki *et al*, 2000). Generally, gastric phenotype carcinomas are considered to have greater invasiveness and metastatic potential than intestinal phenotype carcinomas (Endo *et al*, 1999; Koseki *et al*, 1999; Yoshino *et al*, 1999; Kabashima *et al*, 2002; Tajima *et al*, 2002). In this study, the incidence of reduced or lost Fhit expression in G-type early gastric neoplasias (61.1%) was significantly higher than that in GI-type (28.6%) or I-type (12.8%) early gastric neoplasias. Capuzzi *et al* (2000) reported that the absence of Fhit protein correlated with the progression of gastric carcinomas, higher histological grade and poorer prognosis. Furthermore, MMR-deficient mouse cell lines show increased chromosome fragility (Turner *et al*, 2002). Therefore, combined analyses of phenotypic expression and Fhit expression by immunohistochemistry could be useful methods for evaluating the malignant potential of gastric neoplasias. In addition, gastric phenotype tumours were associated with a loss of Mlh1 expression. In terms of the relationship between genetic alterations and phenotypes of differentiated-type carcinomas, Endo *et al* (1999) reported that MSI was detected more frequently in gastric phenotype carcinomas than in intestinal phenotype carcinomas, which is consistent with the present results. Furthermore, we indicated that the incidence of gastric phenotype in gastric adenomas was significantly lower than that in intramucosal carcinomas. This result was also consistent with a previous report (Tsukashita *et al*, 2001).

In conclusion, we demonstrated reduced Fhit expression in early-stage gastric tumorigenesis, and showed that it was associated with a loss of Mlh1 protein expression and gastric phenotype. Studies that explore the correlation between particular molecular mechanisms and phenotypic expression in neoplasias may offer new insight into gastric carcinogenesis, cancer treatment and feasible chemopreventive pathways.
